# Genome-wide association study for growth traits with 1066 individuals in largemouth bass (*Micropterus salmoides*)

**DOI:** 10.3389/fmolb.2024.1443522

**Published:** 2024-09-25

**Authors:** Wei Han, Ming Qi, Kun Ye, Qiwei He, Dinaer Yekefenhazi, Dongdong Xu, Fang Han, Wanbo Li

**Affiliations:** ^1^ Key Laboratory of Healthy Mariculture for the East China Sea, Ministry of Agriculture and Rural Affairs, Jimei University, Xiamen, China; ^2^ Zhejiang Fisheries Technical Extension Center, Hangzhou, China; ^3^ Key Lab of Mariculture and enhancement of Zhejiang Province, Zhejiang Marine fisheries Research institute, Zhoushan, China

**Keywords:** GWAS, growth traits, largemouth bass, IGF1, breeding

## Abstract

The largemouth bass is a native species of North America that was first introduced to mainland China in the 1980s. In recent years, it has been extensively farmed in China due to its high meat quality and broad adaptability. In this study, we collected growth trait data from 1,066 largemouth bass individuals across two populations. We generated an average of approximately 7× sequencing coverage for these fish using Illumina sequencers. From the samples, we identified 2,695,687 SNPs and retained 1,809,116 SNPs for further analysis after filtering. To estimate the number of genome-wide effective SNPs, we performed LD pruning with PLINK software and identified 77,935 SNPs. Our GWAS revealed 15 SNPs associated with six growth traits. We identified a total of 24 genes related to growth, with three genes—*igf1*, *myf5*, and *myf6*—directly associated with skeletal muscle development and growth, located near the leading SNP on chromosome 23. Other candidate genes are involved in the development of tissues and organs or other physiological processes. These findings provide a valuable set of SNPs and genes that could be useful for genetic breeding programs aimed at enhancing growth in largemouth bass.

## 1 Introduction

Largemouth bass (*Micropterus salmoides*) is a freshwater fish native to rivers and lakes in America (Bai et al., 2008). Due to its strong adaptability, high meat quality, and absence of intermuscular bones, largemouth bass is now extensively farmed in China. First introduced to mainland China in 1983, its production has recently exceeded 700,000 tons (https://www.yearbookchina.com/downsoft-n3020013079.html), making it a crucial species in Chinese freshwater aquaculture (Xie et al., 2020).

Growth is a critical trait in aquaculture species due to its significant economic implications, and fast-growing species are prime targets for improvement (Li et al., 2017). Key growth traits, including body weight (BW), body length (BL), body height (BH), body thickness (BT), caudal peduncle length (CPL), caudal peduncle height (CPH), and condition factor (K) (Li et al., 2017; de Vries et al., 2020), are used to describe growth. These quantitative traits are influenced by polygenes with minor effects, environmental factors, and interactions between genes and the environment (Wang et al., 2006). According to Li et al. (2017), growth trait variation has a strong genetic basis and can be enhanced through phenotypic selection, with genetic gain accelerated by marker-assisted selection.

Genome-wide association study (GWAS) are now the standard method for identifying associations between single nucleotide polymorphisms (SNPs) and phenotypic traits at the whole-genome level, based on linkage disequilibrium (LD) (Visscher et al., 2012). By 2017, over 3,000 human GWAS had investigated more than 1,800 diseases and traits, revealing thousands of SNP associations (Buniello et al., 2019). GWAS has been widely applied to study genetic mechanisms of human diseases and, in aquaculture, to explore growth, disease resistance, meat quality traits, and behavioral traits in species such as Atlantic salmon, catfish, rainbow trout, and carp. For instance, Sodeland et al. (2013) used 5,650 SNPs to identify genetic variations related to fillet fat content and firmness in Norwegian Atlantic salmon, while Tsai et al. (2015) showed that multiple gene loci (*pomt1, myh9, gapdh, notch3, etc.*) influence growth traits in juvenile farmed Atlantic salmon through high-density SNP arrays. Wan et al. (2018) conducted a GWAS on n-3 highly unsaturated fatty acids (HUFA) and eviscerated weight (EW) in the large yellow croaker, identifying three candidate regions for each trait. Zhou et al. (2022) used 5,205 high-quality SNPs to perform a GWAS on six growth traits in 160 mandarin fish (*Siniperca chuatsi*), detecting 11 significant and 37 suggestive SNPs. Huang et al. (2022) identified candidate SNPs and genes associated with body length and weight in 125 female yellow catfish (*Pelteobagrus fulvidraco*), finding one BL-related SNP and three BW-related SNPs significantly associated with these traits.

To date, research on largemouth bass has focused on its distribution, migration, and growth performance with nutrients like chlorogenic acid (Yin et al., 2021). High-density linkage maps for largemouth bass have been constructed (Dong et al., 2019), and recent studies have mapped the sex determination region (He et al., 2022; Wen et al., 2022). However, GWAS studies on growth traits in largemouth bass have not yet been reported. This study aims to identify candidate genes or mutations associated with growth traits in largemouth bass, which could be leveraged for genetic breeding to enhance future yields.

## 2 Materials and methods

### 2.1 Ethics statement

The sample collection of largemouth bass and experiments in this study were approved by the Animal Care and Use Committee of Jimei University (No. JMU202003019).

### 2.2 Sample collection and phenotypic traits measurements

In October 2020 and November 2021, a total of 1,066 largemouth bass were collected from Dongjing Biotechnology Co., Ltd. in Longyan, Fujian Province, China, 688 fish in October 2020 (batch 1) and 378 fish in November 2021 (batch 2). Pectoral fins were sampled from each fish and preserved in 75% ethyl alcohol. We collected phenotypic data on several growth traits, including body length (BL), body weight (BW), body thickness (BT), body height (BH), caudal peduncle length (CPL), and caudal peduncle height (CPH) for each individual. Differences between males and females were assessed using Fisher’s exact test. The fish collected in October 2020 were dissected to determine their phenotypic sex through visual examination of the gonads. For the fish collected in November 2021, sex was determined using a sex-specific marker (He et al., 2022).

### 2.3 Genome sequencing and SNP calling

Genomic DNA was extracted from the pectoral fin using the FastPure® Cell/Tissue DNA Isolation Mini Kit (Vazyme, Nanjing, China). The DNA was sheared and end-repaired, and then ligated with Illumina sequencing adapters to construct sequencing libraries. The libraries were quantified, pooled, and sequenced with 150-bp paired-end mode on a NovaSeq X platform at Novogene Co., Ltd. (Beijing, China). In total, 688 individuals of batch 1 and 378 individuals of batch 2 were sequenced.

After filtering out reads containing adaptor sequences or lower quality bases using Trimmomatic (v.0.36) (Bolger et al., 2014), we aligned the clean sequencing reads to the reference genome of largemouth bass (GenBank accession GCA_019677235.1) (Wen et al., 2022) using BWA (v 0.7.17) (Li and Durbin, 2009). We then sorted and indexed the alignment using Samtools (v1.11) (Danecek et al., 2021) and removed PCR duplicates with the Genome Analysis Toolkit (GATK, v4.2.0) (McKenna et al., 2010; DePristo et al., 2011). HaplotypeCaller function of GATK (v 4.2.0.0) was used to identify SNPs across all samples. PLINK (v1.9) (Yang et al., 2012) was used to filter the SNPs with following parameters: individual missing (geno) < 0.2, Hardy-Weinberg equilibrium (hwe) < 1e-5, minor allele frequency (maf) > 0.05, and SNPs missing rate <0.2. To impute missing sites, we used STITCH (v.1.6.6) with the parameters “nGen = 40, K = 30, ni = 30,” which were most suitable for our data (Davies et al., 2016).

### 2.4 Population structure analysis

Principal component analysis (PCA) of the sequenced fish was conducted using PLINK (v1.9) software. The twstats tool from EIGENSTRAT (v6.1.4) (Price et al., 2006) was employed to select significant principal components (PCs) for the GWAS. Additionally, to assess population kinship, we used Genome-wide Complex Trait Analysis (GCTA) software (v1.93.2) (Yang et al., 2011) to construct the genomic relationship matrix using the--make-grm parameter. A sparse genetic relationship matrix (GRM) was generated from the full-dense GRM using the--make-bK-sparse 0.05 parameter, with a cutoff value of 0.05.

### 2.5 Genome-wide association study (GWAS)

Association analysis was performed using the mixed linear model (MLM) of GCTA software (v1.93.2), the phenotypic data underwent normalization for transformation, and the covariates including the batch effect, gender and the results of PCA in the analysis (Jiang et al., 2019).

To determine the genome-wide significance cutoff for our GWAS results, we estimated the number of effective SNPs by pruning SNPs within a 50 kb window with an R^2^ ≥ 0.8 using PLINK (v1.9). We employed a 50-kb sliding window approach with a step size of 25 kb (Xiang et al., 2020; Wang et al., 2022). The Bonferroni correction was applied to establish the significance threshold, with genome-wide significance defined as 0.05 divided by the number of effective SNPs. The false discovery rate (FDR) was calculated using the Benjamini–Hochberg procedure to adjust the significance threshold (Benjamini and Hochberg, 2018). LD analysis plots were generated using LDBlockshow (v1.40) (Dong et al., 2021) and PopLDdecay (v3.41) (Zhang et al., 2019) software.

### 2.6 Heritability analysis

Heritability is defined as the proportion of variance in phenotype explained (PVE) by genetic variations (Young et al., 2022). The genetic relationship matrix (GRM) was constructed using GCTA, and heritability was calculated by a restricted maximum likelihood (REML) analysis with GCTA software. The PVE was calculated as follows (Shim et al., 2015; Young et al., 2022):

SNPPVE=2 * beta^⁢2 * MAF *1−MAF/2 * beta^⁢2 * MAF⁡1−MAF+(se⁡beta)^⁢2 * 2 * N * MAF *1−MAF

Where: N is the sample size, beta is the effect size for the genetic variant of interest, and MAF is the minor allele frequency for the genetic variant of interest, se (beta) is the standard error of effect size for the genetic variant of interest.

## 3 Results

### 3.1 Phenotypic statistic and correlations

**FIGURE 1:**
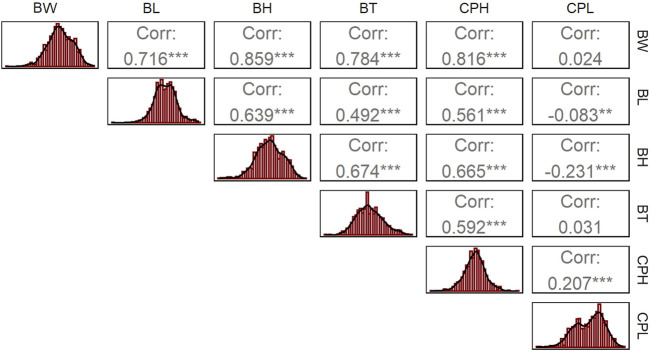
Correlation of each growth trait in largemouth bass. The superscripts of the coefficients indicate the significance of the Pearson correlation: ^*^ denotes *p* < 0.05, ^**^ denotes *p* < 0.01, and ^***^ denotes *p* < 0.001. The histogram on the left of each row shows the distribution of the phenotypic data for each trait, with the *x*-axis representing the trait value and the *y*-axis representing the frequency.

The descriptive statistics results showed that the mean body weight (BW) of largemouth bass was 539.2 ± 111.21 g, with males averaging 548.2 g and females 530.6 g. The mean body length (BL) was 25.15 ± 1.96 cm, with males at 25.08 cm and females at 25.22 cm. The mean body height (BH) was 8.90 ± 0.81 cm, with males at 8.88 cm and females at 8.91 cm. The mean body thickness (BT) was 4.62 ± 0.51 cm, with males at 4.7 cm and females at 4.53 cm. The mean caudal peduncle length (CPL) was 3.17 ± 0.22 cm, with males at 3.15 cm and females at 3.18 cm. The mean caudal peduncle height (CPH) was 5.50 ± 0.66 cm, with males at 5.61 cm and females at 5.39 cm. Phenotypic differences between males and females were statistically significant for all traits (*p*-value range: 0.044–1.35e-08, Fisher’s exact test) except for CPH (*p*-value: 0.077) (Table 1). Correlation analysis revealed significant correlations between all growth traits (*p* < 0.01), except between CPL and BW and BT (Figure 1).

**TABLE 1:** Descriptive statistics of phenotypic data in largemouth bass.

Trait	Number	Mean	SD	Mean in males	Mean in females	*p*-value
BW (g)	1066	539.2	111.21	548.2	530.6	0.019
BL (cm)	1066	25.15	1.96	25.08	25.22	0.044
BH (cm)	1066	8.90	0.81	8.88	8.91	1.35e-08
BT (cm)	1064	4.62	0.51	4.7	4.53	0.002
CPH (cm)	1066	3.17	0.22	3.15	3.18	0.077
CPL (cm)	1066	5.50	0.66	5.61	5.39	2.40e-05

### 3.2 Sequencing and genotyping

We obtained a total of 4.9 TB of clean data through next-generation sequencing. The average sequencing depth for the samples was 6.9 × , with 10 samples sequenced at 11.1-12.3 × coverage. We used STITCH to impute the missing genotypes, achieving an imputation accuracy of 0.92, based on an assessment with the 10 high-coverage samples. After variant calling with GATK, we identified 2,695,687 SNPs. Following quality control, 685,037 variants were removed due to minor allele frequency thresholds (--maf), no individuals were removed due to missing genotype data (--mind), 124,328 variants were removed due to missing genotype data (--geno), and 77,156 variants were removed due to Hardy-Weinberg equilibrium exact test (--hwe). Ultimately, we retained 1,809,166 SNPs for subsequent analyses.

### 3.3 Population structure

**FIGURE 2:**
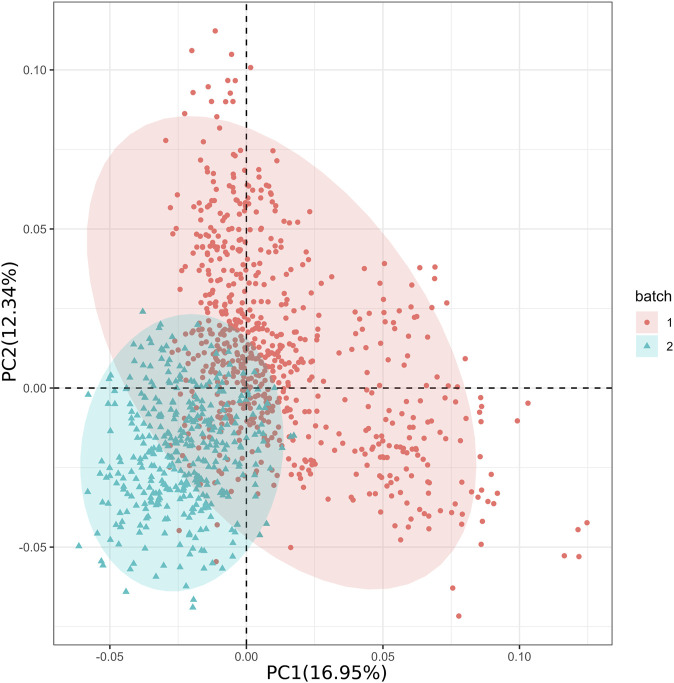
Principal component analysis of the experimental populations.

We used Plink (v1.9) to generate 20 principal components (PCs). After examining the PCA results with EIGENSTRAT’s twstats tool (v6.1.4), we selected 10 PCs with *p*-value <0.05 for further analysis. The PCA outcomes revealed that PC1 accounted for 16.95% of the genetic variation and PC2 accounted for 12.34% (Figure 2). Together, PC1 and PC2 explain a substantial portion of the dataset’s variability. By observing the distribution of these points in the principal component space, we can gain insights into the similarities and differences between different data batches and their trends along the principal components. Additionally, the genetic relationship matrix (GRM) indicated a certain level of population stratification (Supplementary Figure S1). After transforming the GRM into a sparse matrix, no unrelated individuals were removed. Overall, our findings revealed the existence of population stratification among the 1066 individuals collected in two batches.

### 3.4 Genome-wide association study

We used 10 principal components (PCs), sex, and batch effect as covariates in the genome-wide association study. The number of genome-wide effective SNPs was determined by pruning SNPs within 50 kb and with R2 ≥ 0.8 using PLINK (v1.9) with a sliding window approach, resulting in 77,935 effective SNPs. The genome-wide significance threshold was set at 6.42 × 10e-7 (0.05/77,935) based on the number of effective SNPs.

**FIGURE 3:**
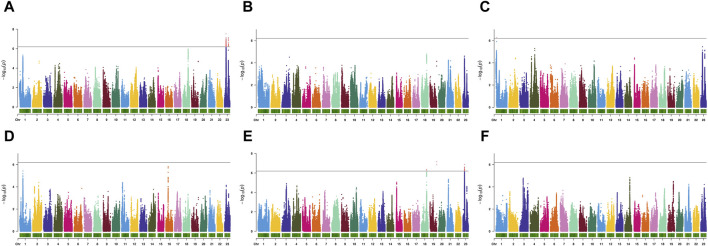
The manhattan plots of genome-wide association analysis of body weight **(A)**, body length **(B)**, body height **(C)**, body thickness **(D)**, caudal peduncle height **(E)**, caudal peduncle length **(F)**. The gray line represents the threshold *p*-value for genome-wide significance (-log10(*p*-value) = 6.19).

Using GCTA software, the estimated heritability for body weight (BW), body length (BL), body height (BH), body thickness (BT), caudal peduncle height (CPH), and caudal peduncle length (CPL) were 0.37, 0.20, 0.44, 0.29, 0.48, and 0.28, respectively (Table 2). According to the GWAS results (Figure 3; Supplementary Figure S2), six SNPs in BW, BH, and CPH reached genome-wide significance. For BW, significant SNPs were located on chromosome 23 at positions 8,370,508 bp and 20,121,867 bp where *p*-value reached 7.52 and 7.14, with phenotypic variance explained (PVE) of 3.35% and 2.64%, respectively (Figure 3A; Table 2). For BH, significant SNPs were located on chromosome 1 at 3,391,027 bp where *p*-value reached 6.28, with PVEs of 2.28%, respectively (Figure 3C; Table 2). For CPH, significant SNPs were located on chromosome 19 at 33,623,024 bp, on chromosome 23 at 8,414,614 bp, and on chromosome 18 at 26,787,765 bp where *p*-value reached 7.15, 6.87 and 6.37, with PVEs of 3.06%, 2.99%, and 2.85%, respectively (Figure 3E; Table 2). In addition, we found the false discovery rate (FDR) results were consistent with the pruning SNPs overall (Table 2).

**TABLE 2:** Information of the significant SNPs.

Trait	Chr	SNP ID	n	h^2^	Ref	Alt	− log10(*p*-value)	MAF	PVE (%)	Related gene
BW	23	23_8370508	1059	0.37	T	C	7.52	0.12606	3.35	*rerg, jup, etv6, lrig3*
	23	23_20121867	1066		A	G	7.14	0.183597	2.64	*myf6, myf5, igf1*
BL	23	23_7805143	1018	0.20	A	G	4.60	0.103617	1.89	*ntf3*
	18	18_26454618	1066		C	A	4.79	0.410831	2.06	null
	21	21_7362633	1066		T	C	4.24	0.184701	1.5	*homer, znf703*
BH	1	1_3391027	1037	0.44	T	G	6.28	0.460463	2.28	*cavin4b*
	23	23_8381853	983		G	T	5.44	0.0942829	2.29	The same with BW
BT	16	16_11727052	1064	0.29	T	C	5.81	0.0878759	2.4	*prdm16, edn3b*
	1	1_15860038	1033		A	G	5.42	0.279768	2.29	*shisa2, flt3, flt1*
CPL	14	14_25230882	1065	0.28	A	G	4.83	0.421596	2.09	*igf2r*
	3	3_17128930	1071		G	A	4.73	0.470121	2.06	*nhsl2, irs2*
	19	19_28093275	1070		C	A	4.48	0.0752336	1.64	*col22a1, paqr7a, kdf1b*
CPH	19	19_33623024	972	0.48	C	T	7.15	0.0704733	3.06	null
	23	23_8414614	1027		A	C	6.87	0.100338	2.99	null
	18	18_26787765	1065		C	T	6.37	0.412207	2.85	*cdk5, fev*

Additionally, we screened SNPs for growth traits of largemouth bass that reached suggestive significance (Table 2). We identified eight SNPs for BL, BT, and CPL traits in the GWAS results. For BL, significant SNPs were located on chromosomes 23, 18, and 21, with PVEs of 1.89%, 2.06%, and 1.5%, respectively (Figure 3B; Table 2). For BT, significant SNPs were located on chromosomes 16 and 1, with PVEs of 2.4% and 2.29%, respectively (Figure 3D; Table 2). For CPL, significant SNPs were located on chromosomes 14, 3, and 19, with PVEs of 2.09%, 2.06%, and 1.64%, respectively (Figure 3F; Table 2).

We investigated candidate genes associated with growth traits near the top SNPs and selected these genes based on linkage disequilibrium (LD) blocks surrounding the top SNPs (see Supplementary Figure S4). Following each of SNP, we ultimately identified 24 candidate genes related to growth traits (Table 2), and the gene function annotations were obtained using the ZFIN (https://zfin.org/), Alliance of Genome Resources (www.alliancegenome.org), and GeneCards (https://www.genecards.org/) web platforms.

For body weight (BW) and caudal peduncle height (CPH) traits, we identified four genes on chromosome 23: *rerg*, *jup*, *etv6*, and *lrig3*. The *rerg* gene inhibits cell proliferation (Finlin et al., 2001). The *jup* gene is involved in alpha-catenin and cadherin binding activities (Boutillon et al., 2022). The *etv6* gene plays a role in embryonic hemopoiesis (De Braekeleer et al., 2012). The *lrig3* gene may be involved in craniofacial and inner ear morphogenesis during embryonic development (Abraira et al., 2010). Additionally, for BW, we identified three genes around the top SNP on chromosome 23: *myf6*, *myf5*, and *igf1*. The *myf6* gene is important for skeletal muscle tissue development (Wei et al., 2024). The *myf5* gene is involved in mesoderm development, muscle organ development, and neural crest cell development (Wei et al., 2022). The *igf1* gene exhibits insulin-like growth factor receptor binding activity, contributing to dorsal and ventral pattern formation and positive regulation of cell proliferation (Duran et al., 2022).

For body length (BL) traits, we identified *ntf3* on chromosome 23. The *ntf3* gene is predicted to have growth factor activity and nerve growth factor receptor binding activity, and is involved in negative regulation of programmed cell death and peripheral nervous system development (Cacialli and Lucini, 2022). Additionally, two genes on chromosome 21 related to BL were highlighted: *homer* and *znf703*. The *homer* gene is involved in muscle structure development (Lin et al., 2013). The *znf703* gene participates in cilium assembly and embryonic camera-type eye morphogenesis (Nakamura et al., 2008).

For body height (BH) traits, we identified the gene *cavin4b* on chromosome 1. The *cavin4b* gene is involved in T-tubule organization and skeletal muscle fiber development (Lo et al., 2021).

For body thickness (BT) traits, we identified two genes on chromosome 16: *prdm16* and *edn3b*. The *prdm16* gene is involved in cardiac muscle cell proliferation, embryonic cranial skeleton morphogenesis, and hematopoietic stem cell differentiation (Shull et al., 2022). The *edn3b* gene plays a role in melanocyte differentiation and pigment cell development (Krauss et al., 2014). Additionally, we identified three genes on chromosome 1 for BT: *shisa2*, *flt3*, and *flt1*. The *shisa2* gene is involved in negative regulation of fibroblast growth factor receptor signaling (Katoh and Katoh, 2005). The *flt3* gene is associated with macrophage differentiation and myeloid cell development (Peng et al., 2015). The *flt1* gene regulates tip cell formation and arterial branching morphogenesis (Krueger et al., 2011).

For caudal peduncle length (CPL) traits, we identified *igf2r* on chromosome 14. We also identified two genes on chromosome 3 related to CPL: *nhsl2* and *irs2*. The *nhsl2* gene is predicted to be involved in cell differentiation (Brooks et al., 2010). The *irs2* gene is predicted to have insulin receptor binding activity (Zasu et al., 2022). Additionally, three genes on chromosome 19 were identified for CPL: *col22a1*, *paqr7a*, and *kdf1b*. For caudal peduncle height (CPH) traits, we identified two genes on chromosome 18: *cdk5* and *fev*. The *cdk5* gene plays a role in nervous system development (Huang et al., 2019). The *fev* gene is involved in hematopoietic stem cell differentiation (Wang et al., 2013).

## 4 Discussion

Improving the growth of largemouth bass is a key objective in the industry, yet effective breeding methods for this species have been lacking. In this study, we utilized genome-wide association studies (GWAS) to identify chromosomal regions associated with growth traits in largemouth bass. Generally, growth traits are complex and influenced by minor genetic factors. Our analysis revealed 24 candidate genes related to growth, including genes involved in growth factors, myogenic factors, neurotrophic factors, and other growth-related functions.

False negatives can arise in GWAS when multiple hypothesis testing methods are employed to establish statistical significance. The two commonly used methods are the Bonferroni correction and the false discovery rate (FDR). However, overly conservative thresholds can lead to a high rate of false negatives. Initially, we applied the Bonferroni correction to set a significance threshold of 2.76e-8 (-log10(*p*-value) = 7.5), calculated as 0.05 divided by the number of tested SNPs (1,809,166). And no SNPs with high *p*-values met the significance criteria for any traits. To refine the significance level for our GWAS results, we focused on effective SNPs by pruning those based on high linkage disequilibrium (LD) to identify SNPs representing high LD values (Xiang et al., 2020; Wang et al., 2022). We determined a genome-wide significant threshold of 6.42e−7 (0.05 divided by the number of effective SNPs). Using this threshold, we identified 12 genes associated with specific growth traits. For the remaining growth traits that did not meet the significant threshold, we selected lead SNPs based on the *p*-values and clustering of SNPs.

Based on phenotypic correlations, body weight (BW) was found to be highly correlated with both body height (BH) and chest perimeter (CPH). Our GWAS results mapped these traits to two SNP clusters located on chromosome 23. Within these regions, we identified a total of six genes. BW and BH shared a common gene, while CPH was associated with one additional gene compared to BW and BH. The identified genes include *rerg*, *jup*, *cd63*, *etv6*, and *lrig3*.

Additionally, the lead SNPs for BW, BH, and CPH were found in another SNP cluster on chromosome 23, specifically at position 20,121,867. In this cluster, we identified three genes: *myf6*, *myf5*, and *igf1*. For body length (BL), the lead SNP, which did not reach the significant threshold on chromosome 23, was located at position 7,805,143. This region was associated with the *ntf3* gene.

Previous studies have demonstrated that growth traits are controlled by multiple genes. Growth factors, which are polypeptides regulating cellular behaviors positively or negatively, play crucial roles in development (Junjie and Shengjie, 2019). Our study identified four key genes directly related to growth and muscle development: insulin-like growth factor I (*igf1*), cation-independent mannose-6-phosphate receptor (*igf2r*) (also known as the insulin-like growth factor 2 receptor), myogenic factor 5 (*myf5*), and myogenic factor 6 (*myf6*).

The growth hormone (GH)/insulin-like growth factor-I (IGF-1) endocrine axis is crucial for regulating growth in fish. IGF-1, produced in response to pituitary GH stimulation of the liver, mediates the growth-promoting effects of GH (Moriyama et al., 2000; Reinecke, 2010). Insulin-like growth factors 1 and 2 have also been linked to testicular germ cell proliferation and apoptosis in fish reproduction (Moreira et al., 2020), and IGF-1 has been shown to regulate muscle growth through integrative transcriptome and microRNAome analyses in Pacu (*Piaractus mesopotamicus*) (Duran et al., 2022). A transcriptomic study of largemouth bass suggested that the GH-IGF-1 axis and its signaling pathway are associated with muscle growth development (Li et al., 2017). This study observed that IGF2R was downregulated and IGFBP1 was upregulated in fast-growing largemouth bass, while IGF2R was upregulated in slow-growing fish (Li et al., 2017). Our investigation also identified the *igf2r* gene on chromosome 14 associated with caudal peduncle length (CPL), predicted to have IGF binding activity and involvement in lysosomal transport. The *igf2r* gene is predicted to have insulin-like growth factor binding activity (Rajeswari et al., 2022). Li et al. (2017) reported increased IGF2R expression in slow-growing largemouth bass. Similarly, Azizi et al. (2016) observed elevated IGF2R expression during myogenesis in gilthead sea bream, suggesting that IGF-2 stimulates muscle growth. In Atlantic salmon, IGF2R transcripts were upregulated during prolonged fasting and downregulated after 7 days of refeeding, indicating that IGF2R may act as a negative regulator for IGF-2 (Abu El-Magd et al., 2014). These findings suggest that both IGF1 and IGF2R play significant roles in the growth of largemouth bass.

Fish meat quality is influenced by the composition and content of nutrients such as amino acids and fatty acids. At the molecular level, the growth and differentiation of fish muscle are regulated by myogenic regulatory factors (MRFs). MRFs affect muscle growth and meat quality (Tan and Du, 2002).

Our study identified two MRFs genes, *myf5* and *myf6*, related to the initiation and development of skeletal muscle. *Myf5* is expressed primarily in satellite cells and muscle spindles (Beauchamp et al., 2000; Zammit et al., 2004), while *myf6* is involved in muscle cell differentiation and has similar functions to MYOG (Lazure et al., 2020). Moghadam et al. (2007) investigated *igf1*, *igf2*, *myf5*, *myf6*, and GRF/PACAP in rainbow trout (*Oncorhynchus mykiss*), Atlantic salmon (*Salmo salar*), and Arctic charr (*Salvelinus alpinus*), demonstrating that *igf1*, *myf5*, and *myf6* are critical for growth in these species.

We also identified genes related to CPL trait: *nhsl2*, *irs2, col22a1*, *paqr7a*, and *kdf1b*. The *nhsl2* gene is predicted to be involved in cell differentiation (Brooks et al., 2010). The *irs2* gene is predicted to have insulin receptor binding activity (Zasu et al., 2022). The *col22a1* gene is involved in embryonic caudal fin morphogenesis, muscle attachment, and fin regeneration (Charvet et al., 2013). The *paqr7a* gene may be involved in oocyte maturation (Wang et al., 2021). The *kdf1b* gene is predicted to regulate epidermal cell division (Shamseldin et al., 2017).

In summary, we have identified significant SNPs and candidate genes associated with growth traits through GWAS. To validate these SNPs, future studies should include growth phenotypes and genotypes from additional largemouth bass cohorts. These SNPs can be employed in genomic selection (GS) to enhance growth in largemouth bass populations, offering a rapid and accurate method for assessing genetic value. Our functional annotations indicate that growth and myogenic factors directly impact the growth and development of largemouth bass, while other candidate genes are involved in tissue and organ development and various physiological processes. This GWAS provides a foundation for the selection and breeding of largemouth bass.

## 5 Conclusion

In this study, we utilized a total of 4.9 Terabytes of clean data from 1066 individuals and obtained 1,809,166 SNPs after variant calling and quality control. Based on this, we found 24 candidate genes (*rerg, jup, etv6, lrig3, myf6, myf5, igf1, ntf3, homer, znf703, cavin4b, prdm16, edn3b, shisa2, flt3, flt1, igf2r, nhsl2, irs2, col22a1, paqr7a, kdf1b, cdk5,and fev*) that are related to the growth traits of largemouth bass, as determined by the Genome-wide association study (GWAS) results. Three genes, namely, *igf1*, *myf5*, and *myf6*, which are directly associated with the development of skeletal muscle and individual growth, were located around the lead SNP on chromosome 23. These findings provide useful information for selection and breeding in largemouth bass.

## Data Availability

The datasets presented in this study can be found in online repositories. The names of the repository/repositories and accession number(s) can be found below: https://www.ncbi.nlm.nih.gov/bioproject, PRJNA748185.
