# Supplementary material for: Genome-wide association study for growth traits with 1066 individuals in largemouth bass (Micropterus salmoides)
Source: Front Mol Biosci. 2024 Sep 25;11:1443522. doi: 10.3389/fmolb.2024.1443522 (PMC11461307; doi:10.3389/fmolb.2024.1443522)
Supplement: Supplementary file 1 [file DataSheet1.PDF]

Supplementary Material

Genome-Wide Association Study for Growth Traits with 1066 Individuals in Largemouth Bass (*Micropterus salmoides*)

Wei Han[1†], Ming Qi[2†], Kun Ye[1], Qiwei He[1], Dinaer Yekefenhazi[1], Dongdong Xu[3], Fang Han[1*], Wanbo Li[1*]

*** Correspondence:**

Fang Han: hanfangyc@jmu.edu.cn
Wanbo Li: li.wanbo@jmu.edu.cn

1 Supplementary Figures

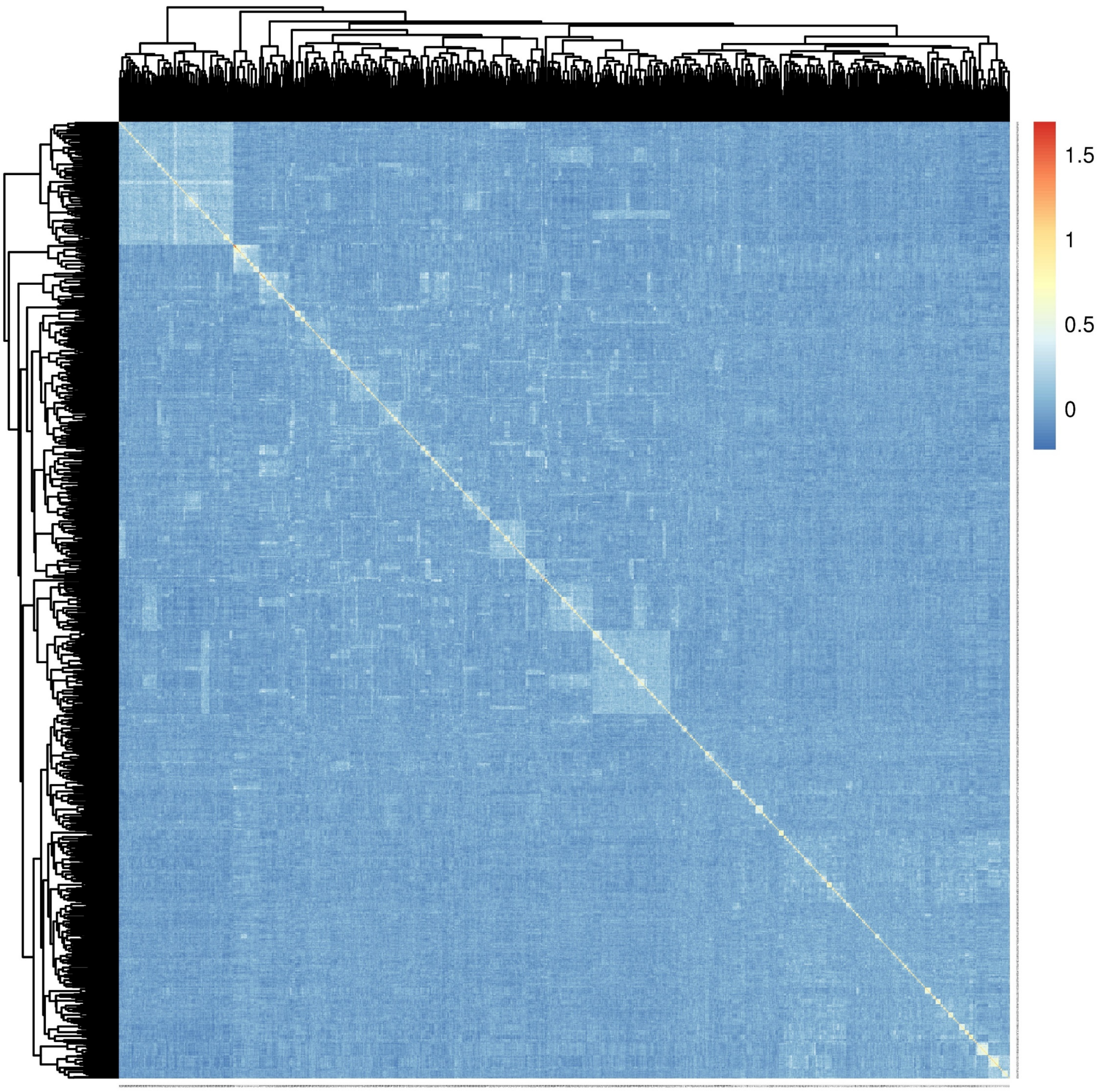

Supplementary Figure 1 Heat map of population genetic relationship of largemouth bass.

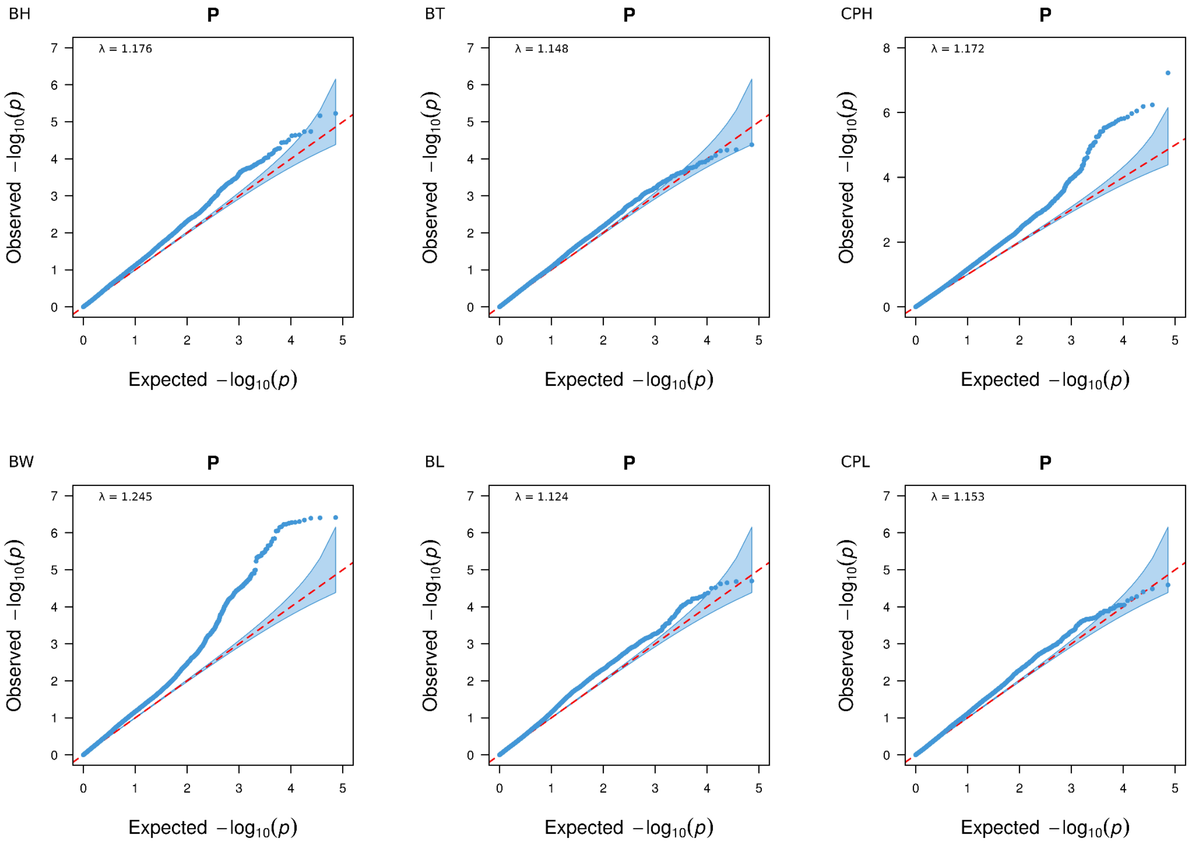

Supplementary Figure 2 QQplot of each trait of GWAS.

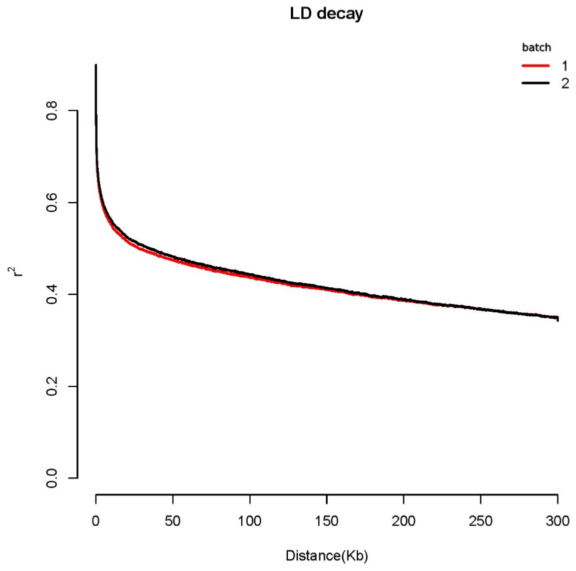

Supplementary Figure 3 LDdecay plot for each batch of largemouth bass.

BW

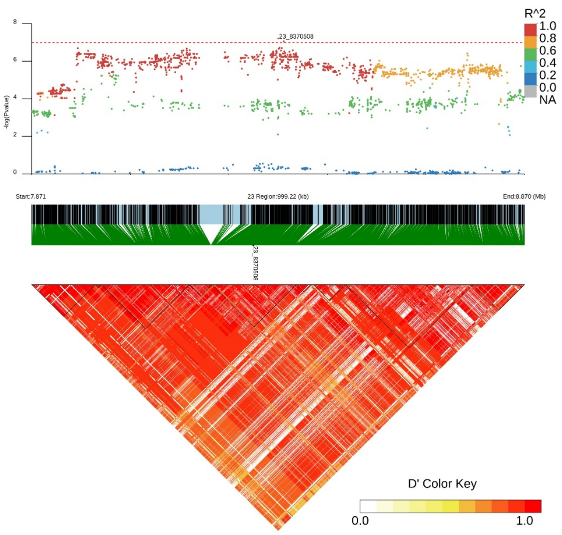
R^2
1.0
0.8
0.6
0.4
0.2
0.0
NA
23_8370508
-log(Pvalue)
Start:7.871
23 Region:999.22 (kb)
End:8.870 (Mb)
D' Color Key
0.0
1.0

BW

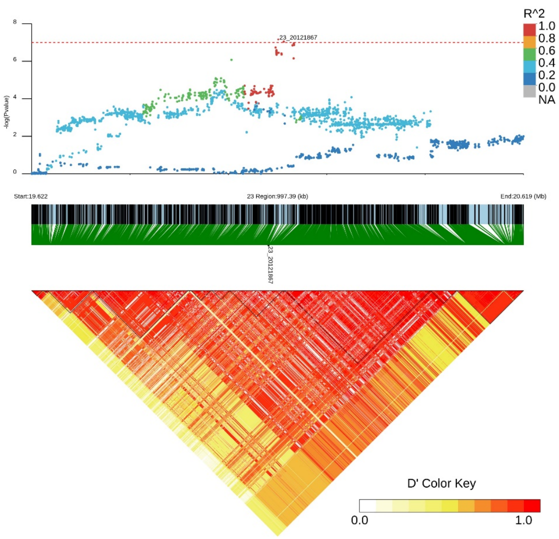
R^2
1.0
0.8
0.6
0.4
0.2
0.0
NA
23_20121867
-log(Pvalue)
Start:19.622
23 Region:997.39 (kb)
End:20.619 (Mb)
D' Color Key
0.0
1.0

BL

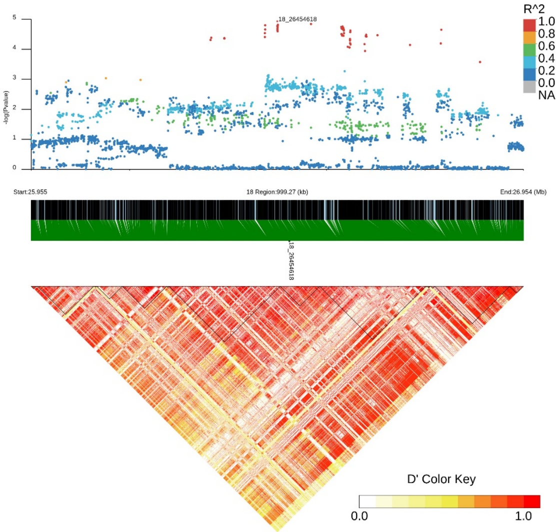

R^2
1.0
0.8
0.6
0.4
0.2
0.0
NA
-log(Pvalue)
18_26454618
Start:25.955
18 Region:999.27 (kb)
End:26.954 (Mb)
D' Color Key
0.0
1.0

BL

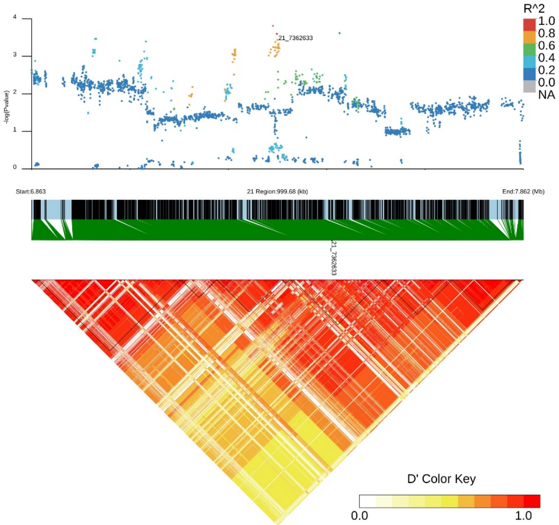

R^2
1.0
0.8
0.6
0.4
0.2
0.0
NA
-log(Pvalue)
21_7362633
Start:6.863
21 Region:999.68 (kb)
End:7.862 (Mb)
D' Color Key
0.0
1.0

BL

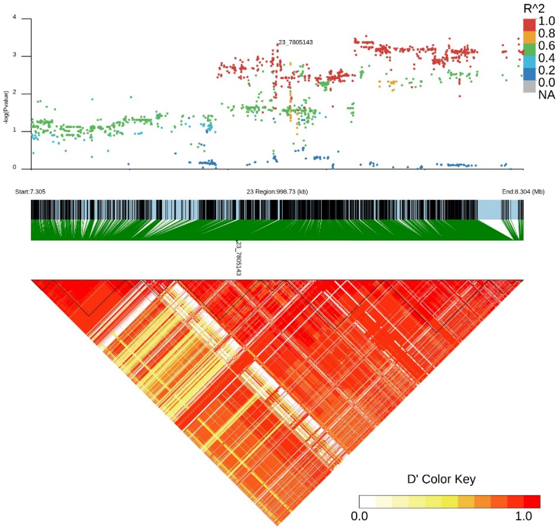

R^2
1.0
0.8
0.6
0.4
0.2
0.0
NA
-log(Pvalue)
23_7805143
Start:7.305
23 Region:998.73 (kb)
End:8.304 (Mb)
D' Color Key
0.0
1.0

BH

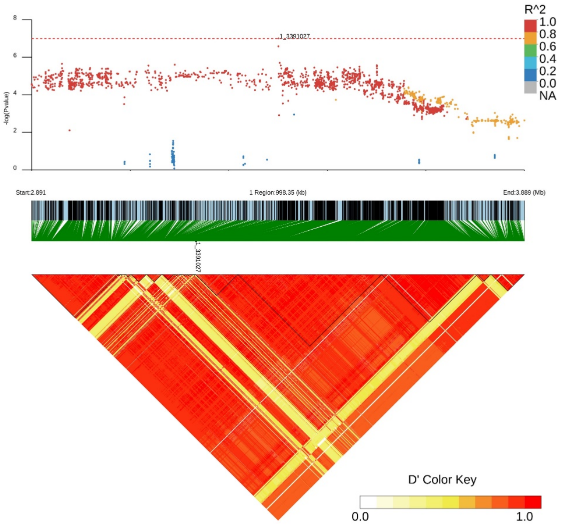

R^2
1.0
0.8
0.6
0.4
0.2
0.0
NA
-log(Pvalue)
1_3391027
Start:2.891
1 Region:998.35 (kb)
End:3.889 (Mb)
D' Color Key
0.0
1.0

BH

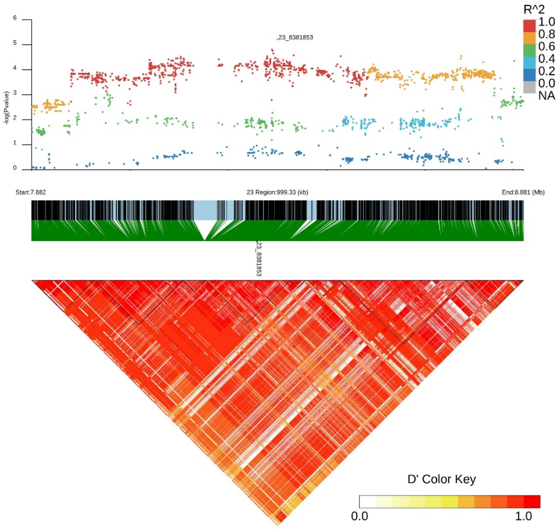

R^2
1.0
0.8
0.6
0.4
0.2
0.0
NA
-log(Pvalue)
23_8381853
Start:7.882
23 Region:999.33 (kb)
End:8.881 (Mb)
D' Color Key
0.0
1.0

BT

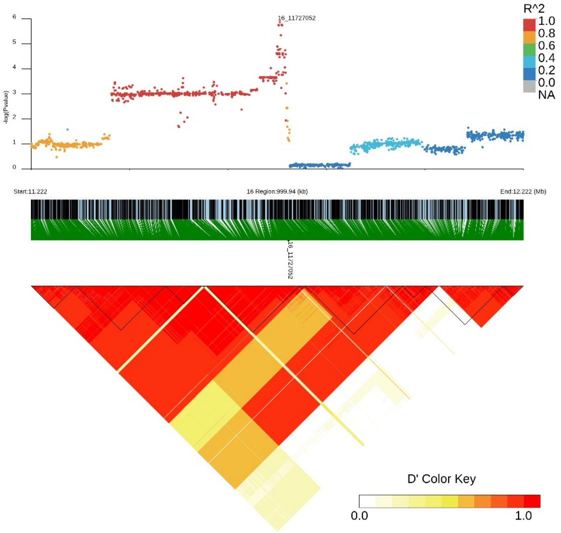

R^2
1.0
0.8
0.6
0.4
0.2
0.0
NA
-log(Pvalue)
16_11727052
Start:11.222
16 Region:999.94 (kb)
End:12.222 (Mb)
D' Color Key
0.0
1.0

BT

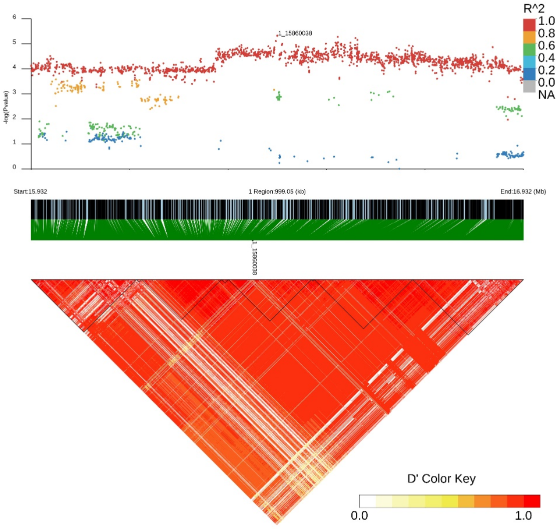

R^2
1.0
0.8
0.6
0.4
0.2
0.0
NA
-log(Pvalue)
1_15860038
Start:15.932
1 Region:999.05 (kb)
End:16.932 (Mb)
D' Color Key
0.0
1.0

CPL

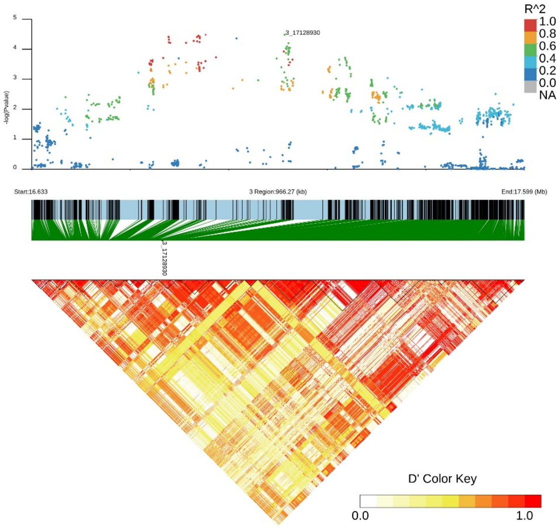
R^2
1.0
0.8
0.6
0.4
0.2
0.0
NA
3_17128930
-log(Pvalue)
Start:16.633
3 Region:966.27 (kb)
End:17.599 (Mb)
D' Color Key
0.0
1.0

CPL

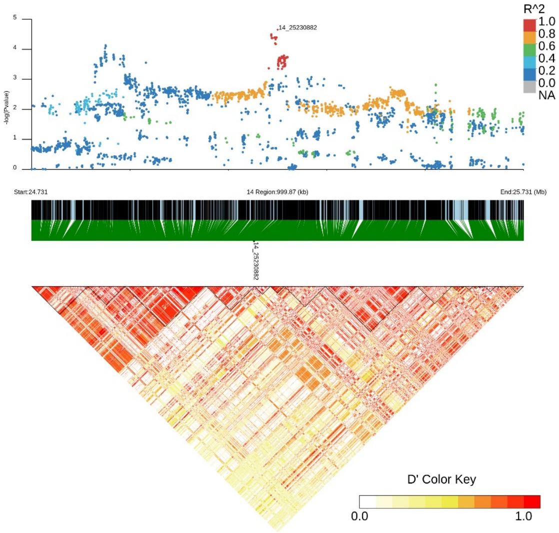
R^2
1.0
0.8
0.6
0.4
0.2
0.0
NA
14_25230882
-log(Pvalue)
Start:24.731
14 Region:999.87 (kb)
End:25.731 (Mb)
D' Color Key
0.0
1.0

CPL

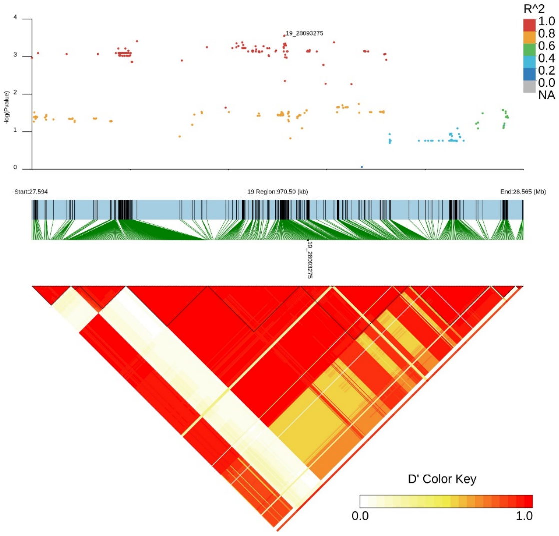
R^2
1.0
0.8
0.6
0.4
0.2
0.0
NA
19_28093275
-log(Pvalue)
Start:27.594
19 Region:970.50 (kb)
End:28.565 (Mb)
D' Color Key
0.0
1.0

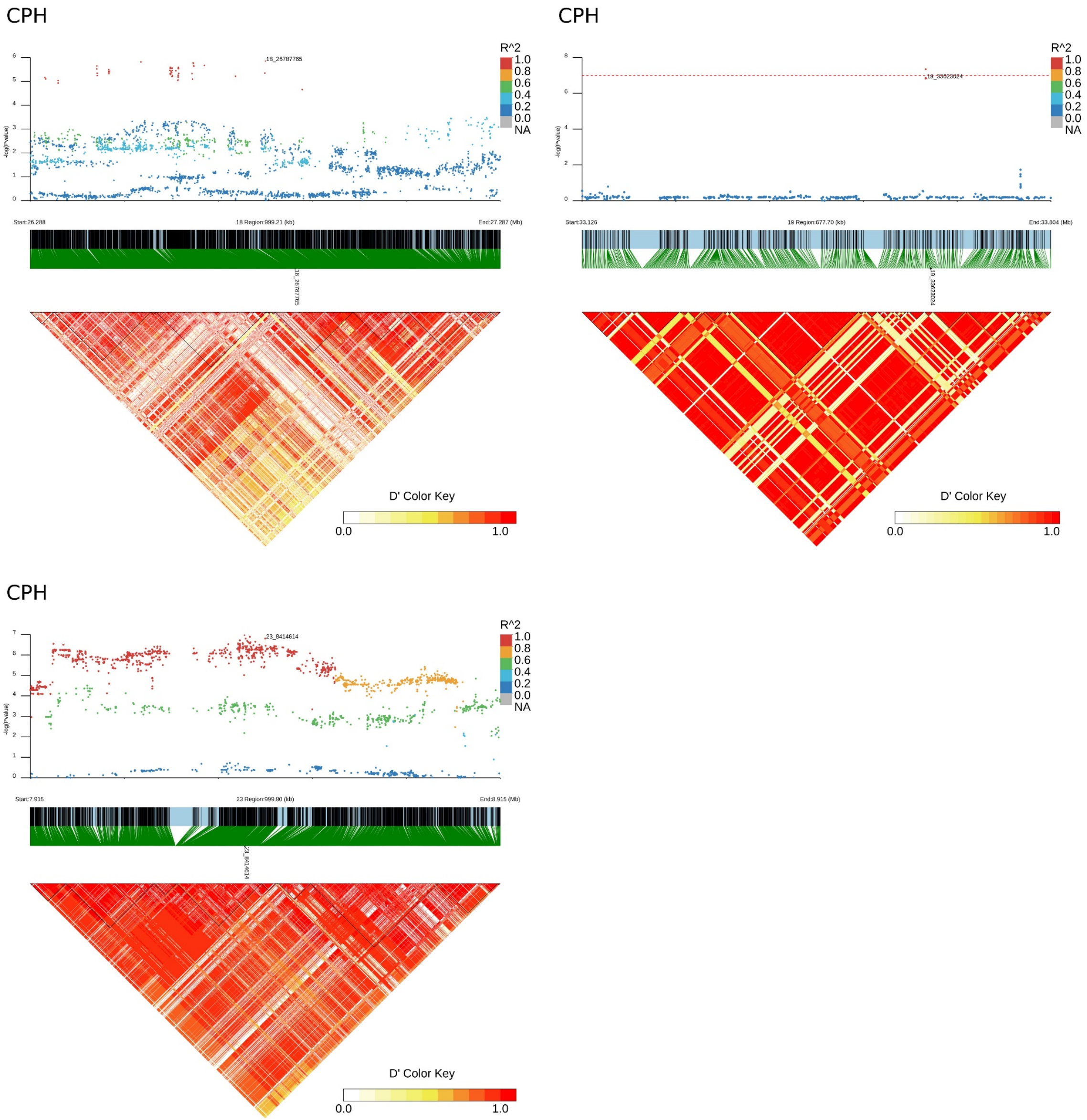

Supplementary Figure S4 LD heatmap.
